# Lamin A safeguards the m^6^A methylase METTL14 nuclear speckle reservoir to prevent cellular senescence

**DOI:** 10.1111/acel.13215

**Published:** 2020-08-19

**Authors:** Jie Zhang, Ying Ao, Zhen Zhang, Yanzhen Mo, Linyuan Peng, Yue Jiang, Zimei Wang, Baohua Liu

**Affiliations:** ^1^ Shenzhen Key Laboratory for Systemic Aging and Intervention National Engineering Research Center for Biotechnology (Shenzhen) Shenzhen University Shenzhen China; ^2^ Department of Biochemistry & Molecular Biology Guangdong Key Laboratory of Genome Stability and Human Disease Prevention School of Basic Medical Sciences Shenzhen University Shenzhen China; ^3^ Shenzhen University‐Friedrich Schiller Universität Jena Joint PhD Program Friedrich Schiller Universität Jena Germany; ^4^ Carson International Cancer Center Shenzhen University Shenzhen China; ^5^ Guangdong Provincial Key Laboratory of Regional Immunity and Diseases School of Basic Medical Sciences Shenzhen University Health Science Center Shenzhen China

**Keywords:** Lamin A, METTL14, METTL3, nuclear speckle

## Abstract

Mutations in *LMNA* gene are frequently identified in patients suffering from a genetic disorder known as Hutchison–Gilford progeria syndrome (HGPS), providing an ideal model for the understanding of the mechanisms of aging. Lamin A, encoded by *LMNA*, is an essential component of the subnuclear domain‒nuclear speckles; however, the functional significance in aging is unclear. Here, we show that Lamin A interacts with the m^6^A methyltransferases, METTL3 and METTL14 in nuclear speckles. Lamin A deficiency compromises the nuclear speckle METTL3/14 reservoir and renders these methylases susceptible to proteasome‐mediated degradation. Moreover, METTL3/14 levels progressively decline in cells undergoing replicative senescence. Overexpression of *METTL14* attenuates both replicative senescence and premature senescence. The data reveal an essential role for Lamin A in safeguarding the nuclear speckle reservoir of the m^6^A methylase METTL14 to antagonize cellular senescence.

## Graphical abstract

Lamin A interacts with the m^6^A methylase METTL14 to ensure the proper localization in the nuclear speckles and protein stability. METTL14 declines in senescent cells, while its overexpression antagonizes cellular senescence.

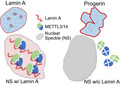

## 1 INTRODUCTION

Lamin A is a major component of nuclear lamina and multiple subnuclear domains; it is first synthesized as a precursor (prelamin A) and then processed by ZMPSTE24 for maturation (Pendas et al., 2002). A *de novo* G608G mutation in *LMNA* gene causes a 50 amino acid truncation of prelamin A. The mutated form of prelamin A, also known as Progerin, is considered to be pathogenic in Hutchinson–Gilford progeria syndrome (HGPS) (Eriksson et al., 2003). Mice lacking *Zmpste24* accumulate prelamin A and develop premature aging features that resemble HGPS (Pendas et al., 2002). Cultured fibroblasts derived from *Zmpste24* ^−/−^ mice and HGPS patients undergo accelerated senescence and apoptosis, attributable to genomic instability, hyperactivation of the p53 pathway and epigenetic changes, etc. (Bridger & Kill, 2004; Krishnan et al., 2011; Liu et al., 2005, 2013; Varela et al., 2005). Although it is extremely rare, HGPS offers an ideal model to understand the mechanisms of aging and age‐related diseases.

Lamins serve as an anchor for proteins that shuttle between the nuclear lamina and the nucleoplasm, and as a platform for RNA metabolism (Ho, Jaalouk, Vartiainen, & Lammerding, 2013; Scaffidi & Misteli, 2008). Lamin A, Lamin B1, and Lamin C1/C2 and multiple proteins that are involved in RNA modification are detectable in the nuclear speckles, also called interchromatin granule clusters (IGCs) (Mintz, Patterson, Neuwald, Spahr, & Spector, 1999). Indeed, N6‐methyladenosine (m^6^A) methylases METTL3 and METTL14 are constantly observed in the nuclear speckles, which form a methyltransferase complex that methylates adenosine residues at the N6 position. This methylation event is the most prevalent internal post‐transcriptional modification to occur on mammalian mRNAs. METTL3 and METTL14 interact with each other via an extensive hydrogen bond network, wherein METTL3 primarily functions as the catalytic core while METTL14 serves as the RNA‐binding platform (Wang et al., 2017).

Here, we aimed to understand the functional relevance of Lamin A‐containing nuclear speckles in cellular senescence. We confirmed Lamin A as an essential component of the nuclear speckles. Further, we found that Lamin A interacts with METTL3/14, thus to ensure their proper localization in the nuclear speckles and protein stability. Most importantly, *METTL14* overexpression prevents cell senescence.

## 2 RESULTS

### 2.1 METTL3/14 interacts with Lamin A in nuclear speckles

**FIGURE 1:**
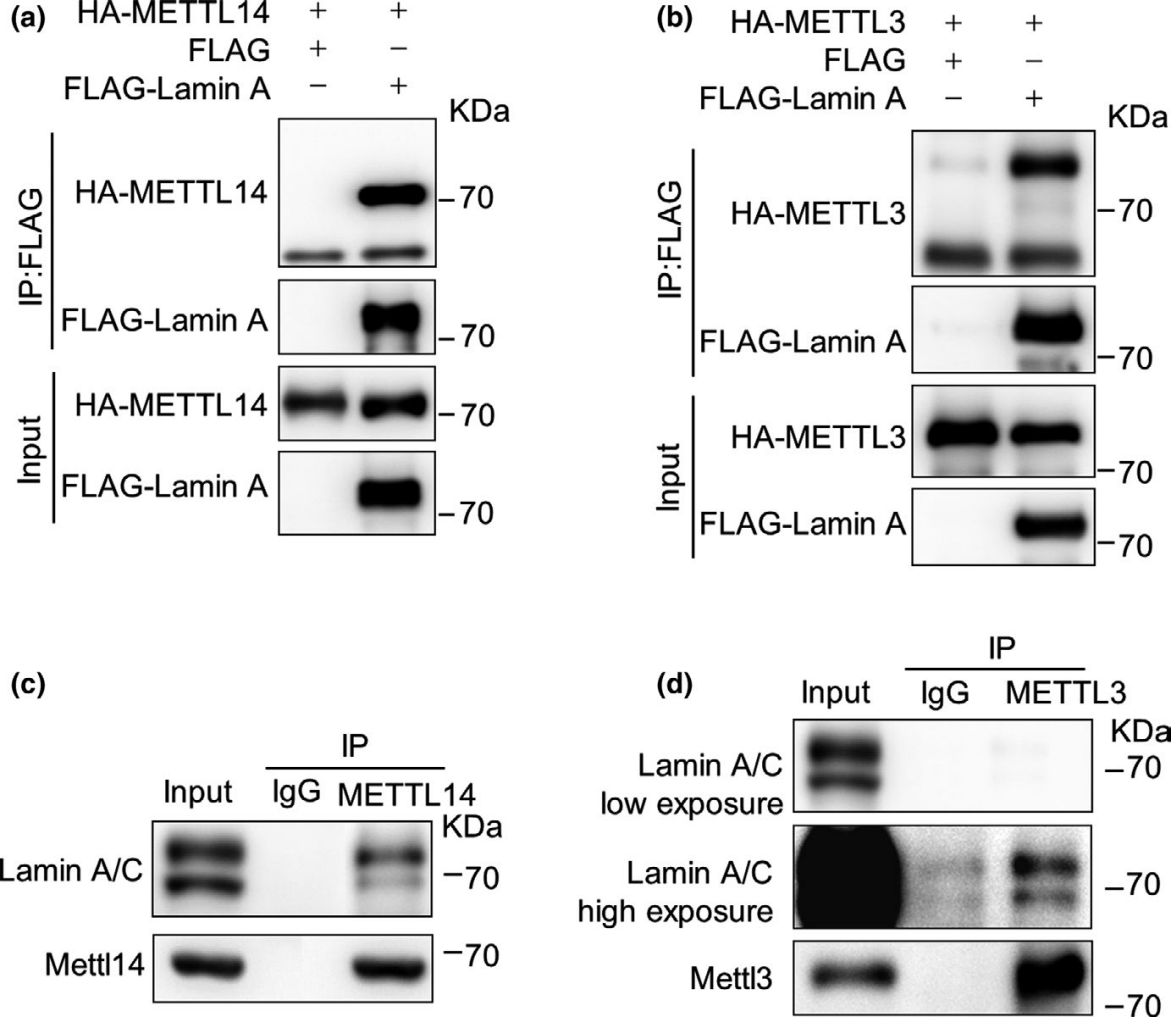
Lamin A interacts with METTL3/14. (a‐b) Co‐immunoprecipitation (Co‐IP) and Western blot analysis of the interactions between FLAG‐Lamin A and HA‐METTL14 (a); and FLAG‐Lamin A HA‐METTL3 (b) in HEK293 cells. (c‐d) Co‐IP and Western blot analysis of the endogenous interaction between Lamin A and METTL14 (c) and Lamin A and METTL3 (d) in MEFs

Nuclear speckles are enriched with RNA‐modifying complex, for example, METTL3/14 m^6^A methyltransferase (Scholler et al., 2018; Spector & Lamond, 2011). Lamin A is also evidenced in the nuclear speckles (Kumaran, Muralikrishna, & Parnaik, 2002). Splicing factor SC‐35 is a maker protein in the nuclear speckles (Klein et al., 2016). Indeed, we noticed that almost all SC‐35 foci were co‐localized with Lamin A and more than half of them were co‐localized with METTL3/14 in wild‐type (WT) mouse embryonic fibroblasts (MEFs) (Figure S1a). In primary human skin fibroblasts (HSFs) co‐transfected with FLAG‐METTL3/14 and DsRed‐Lamin A (LA‐Red), most of the FLAG‐METTL3/14‐positive speckles also expressed LA‐Red (Figure S1b). We thus reasoned that Lamin A might interact with METTL3/14 in the nuclear speckles. To test the hypothesis, we did co‐immunoprecipitation (Co‐IP) in HEK293 cells overexpressing FLAG‐Lamin A with HA‐METTL14 or HA‐METTL3. As shown, HA‐METTL14 and HA‐METTL3 were present in anti‐FLAG‐Lamin A immunoprecipitates from HEK293 cells (Figure 1a,b). In addition, endogenous Lamin A was co‐immunoprecipitated with METTL3 (Figure 1c) and METTL14 (Figure 1d) in MEFs. Together, these results indicate that Lamin A interacts with METTL3/14 in the nuclear speckles.

### 2.2 Lamin A safeguards the proper nuclear localization of METTL3/14

**FIGURE 2:**
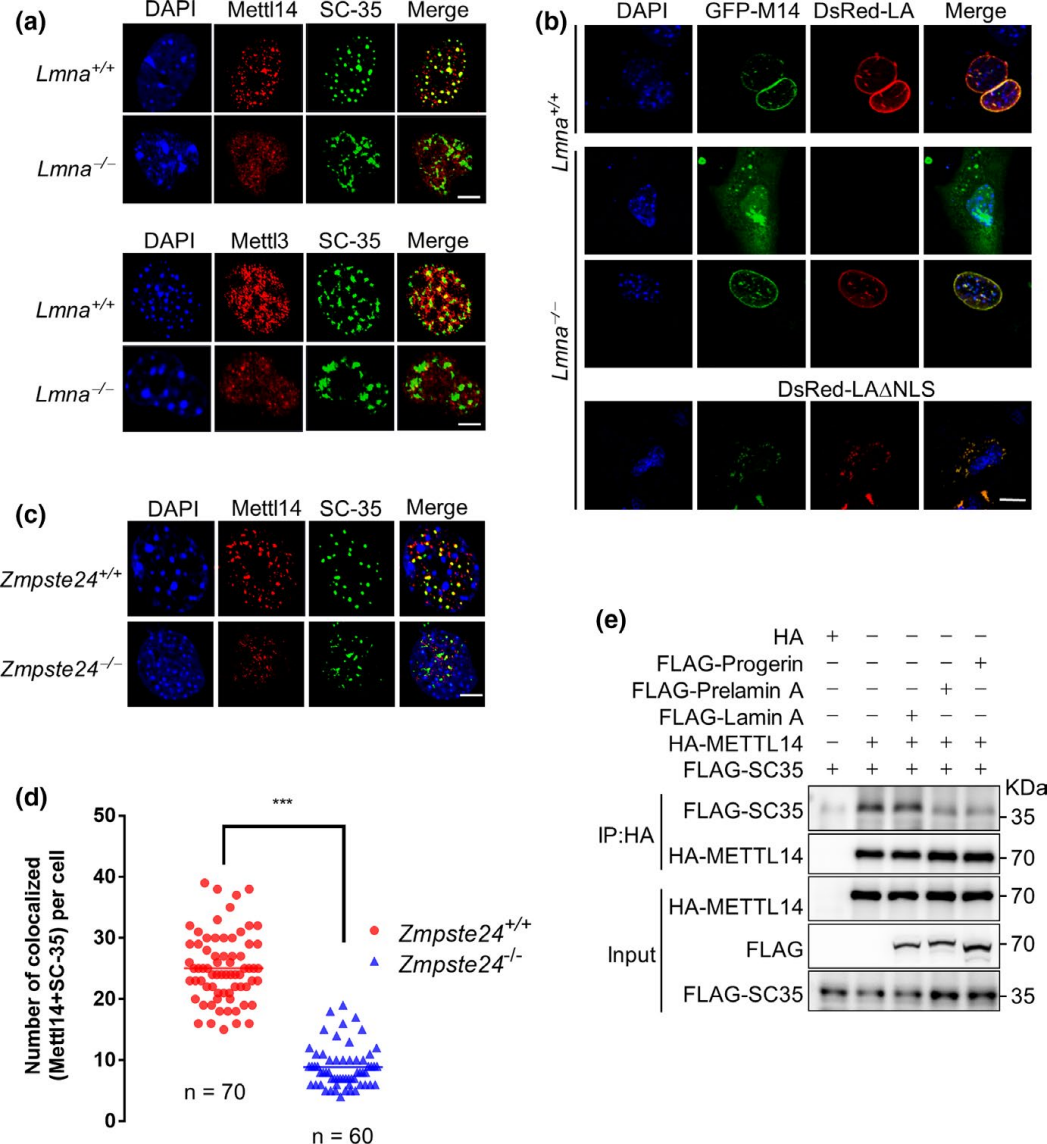
The nuclear localization of METTL3/14 requires Lamin A. (a) Representative immunofluorescence analysis of METTL3, METTL14, and SC35 foci in *Lmna* ^+/+^ and *Lmna* ^−/−^ MEFs. (b) Representative immunofluorescence analysis of GFP‐METTL14 (GFP‐M14) in *Lmna* ^+/+^ and *Lmna* ^−/−^ MEFs with or without DsRed‐Lamin A (Red‐LA). (c) Representative immunofluorescence analysis of METTL14 and SC35 foci in *Zmpste24* ^+/+^ and *Zmpste24* ^−/−^ MEFs. (d) The quantitative analysis of (c). ***p < 0.001. (e) Co‐IP and Western blot analysis of the interaction between SC35 and METTL3/14 in the presence of Lamin A, Progerin, and prelamin A. Scale bars, 10 µm

To examine whether the nuclear speckle localization of METTL3/14 requires Lamin A, we first performed immunofluorescence microscopy to assess the localization of METTL3/14 in *Lmna* ^−/−^ MEFs. We found that SC‐35 foci were visually more diffuse in *Lmna* ^−/−^ MEFs, and almost all of them were negative for METTL3/14 expression (Figure 2a). We then overexpressed GFP‐METTL14 (GFP‐M14) and DsRed‐Lamin A (DsRed‐LA) into *Lmna* ^−/−^ MEFs. As shown, normal METTL14 localization was restored by Lamin A overexpression (Figure 2b). By contrast, this rescue effect was not achieved when these MEFs were reconstituted with a mutant Lamin A protein, in which the Lamin A nuclear localization sequence (NLS) was deleted.

We next examined the localization of METTL14 in *Zmpste24* ^−/−^ MEFs, in which prelamin A accumulates. Interestingly, the number of METTL14 foci that co‐localized with SC35 expression was significantly decreased in *Zmpste24* ^−/−^ MEFs compared with that in WT MEFs (Figure 2c,d). We then performed a Co‐IP experiment between METTL14 and SC35, in the presence of Lamin A, prelamin A and Progerin. The binding of HA‐METTL14 to FLAG‐SC35 was substantially compromised in the presence of prelamin A/Progerin compared to that of Lamin A (Figure 2e). These findings suggest that loss of *Lmna* compromises the proper localization of METTL3/14 in the nuclear speckles and prelamin A/Progerin rather leads to diffuse nuclear distribution of METTL14.

### 2.3 Lamin A abnormalities destabilize METTL3/14

**FIGURE 3:**
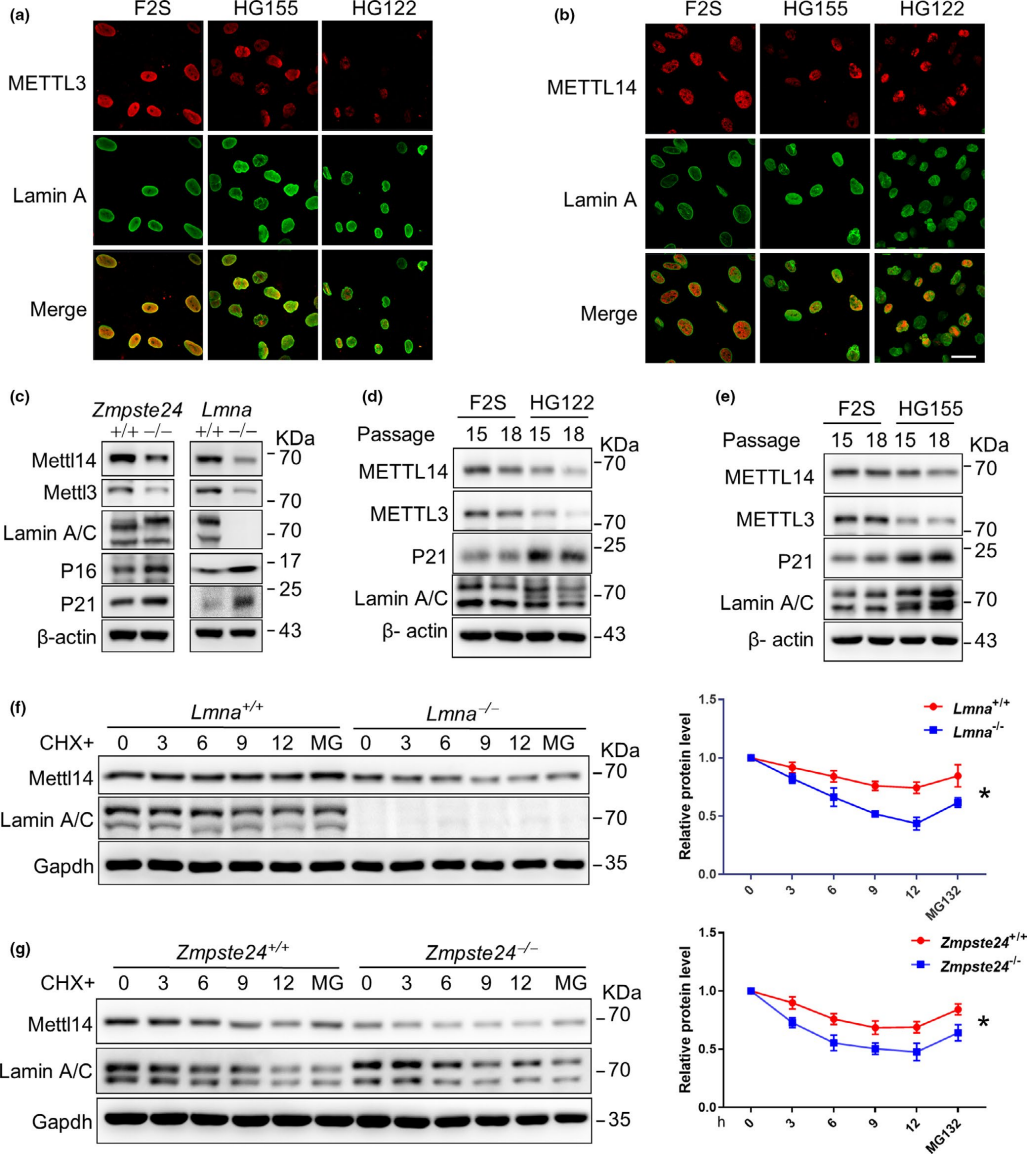
Lamin A abnormality destabilizes METTL14 protein. (a, b) Representative immunofluorescence images showing METTL14/3 and Lamin A expression in skin fibroblasts derived from individual HGPS patients (HG122 and HG155) and normal human skin fibroblasts (F2S). Scale bar, 50 µm. (c) Western blot analysis of METTL3/14 protein levels in *Zmpste24* ^+/+^, *Zmpste24* ^−/−^, *Lmna* ^+/+^, and *Lmna* ^−/−^MEFs. The data represent three independently derived sets of MEFs in separate experiments. (d, e) Western blot analysis of METTL3/14 protein levels in HG122, HG155, and F2S cells at different passages. (f, g) Western blot analysis of METTL3/14 expression in *Lmna* ^−/−^ (f) and *Zmpste24* ^−/−^ (g) MEFs compared with WT cells. Cells were treated with cycloheximide (CHX) and/or MG132 (MG) for indicated time. Quantification was performed by Image J^®^. The data represent the means ± *SEM*. *p < 0.05

We noticed that the levels of METTL3/14 were substantially decreased in *Zmpste24* ^−/−^ and *Lmna* ^−/−^ MEFs (Figure 2a,c). Further, immunofluorescence staining confirmed the reduced METTL3/14 levels in fibroblasts derived from individual HGPS patients (Figure 3a,b). We reasoned that nuclear speckle occupancy might help maintain the expression of METTL3/14. To test the hypothesis, we did Western blotting to examine METTL3/14 protein levels in *Zmpste24* ^−/−^ MEFs, *Lmna* ^−/−^ MEFs, and HGPS cells. Compared with WT, the METTL3/14 levels were obviously reduced in *Lmna* ^−/−^ and *Zmpste24* ^−/−^ MEFs (Figure 3c) despite their mRNA levels being unaffected (Figure S2). METTL3/14 protein levels were also substantially decreased in HGPS cells at different passages compared with that in fibroblasts from healthy individuals (Figure 3d,e). Of note, the level of p21^WIF1^, which is a widely accepted molecular marker of senescence, was negatively correlated with that of METTL3/14 (Figure 3c‐e). This finding supports that METTL3/14 levels decrease in premature senescence.

Given that METTL14 expression seemed to decrease in Lamin A‐deficient cells, we presumed that WT Lamin A might enhance the protein stability of METTL14. Indeed, after the treatment of cycloheximide (CHX) together with or without MG132, which inhibited protein synthesis and proteasome activity, respectively, we found that the protein degradation rate of METTL14 was accelerated in *Zmpste24* ^−/−^, *Lmna* ^−/−^, and *Lmna^G609G^* ^/^ *^G609G^* (Progerin knock‐in) MEFs compared with that in WT MEFs (Figure 3f,g and Figure S3). Moreover, the ubiquitination level of METTL14 was increased in *Zmpste24* ^−/−^ MEFs (Figure S4). Collectively, the data suggest that Lamin A protects METTL14 protein from proteasomal degradation and the absence of Lamin A or the presence of prelamin A induces protein degradation of METTL14.

### 2.4 A decline in METTL14 expression accelerates cellular senescence

**FIGURE 4:**
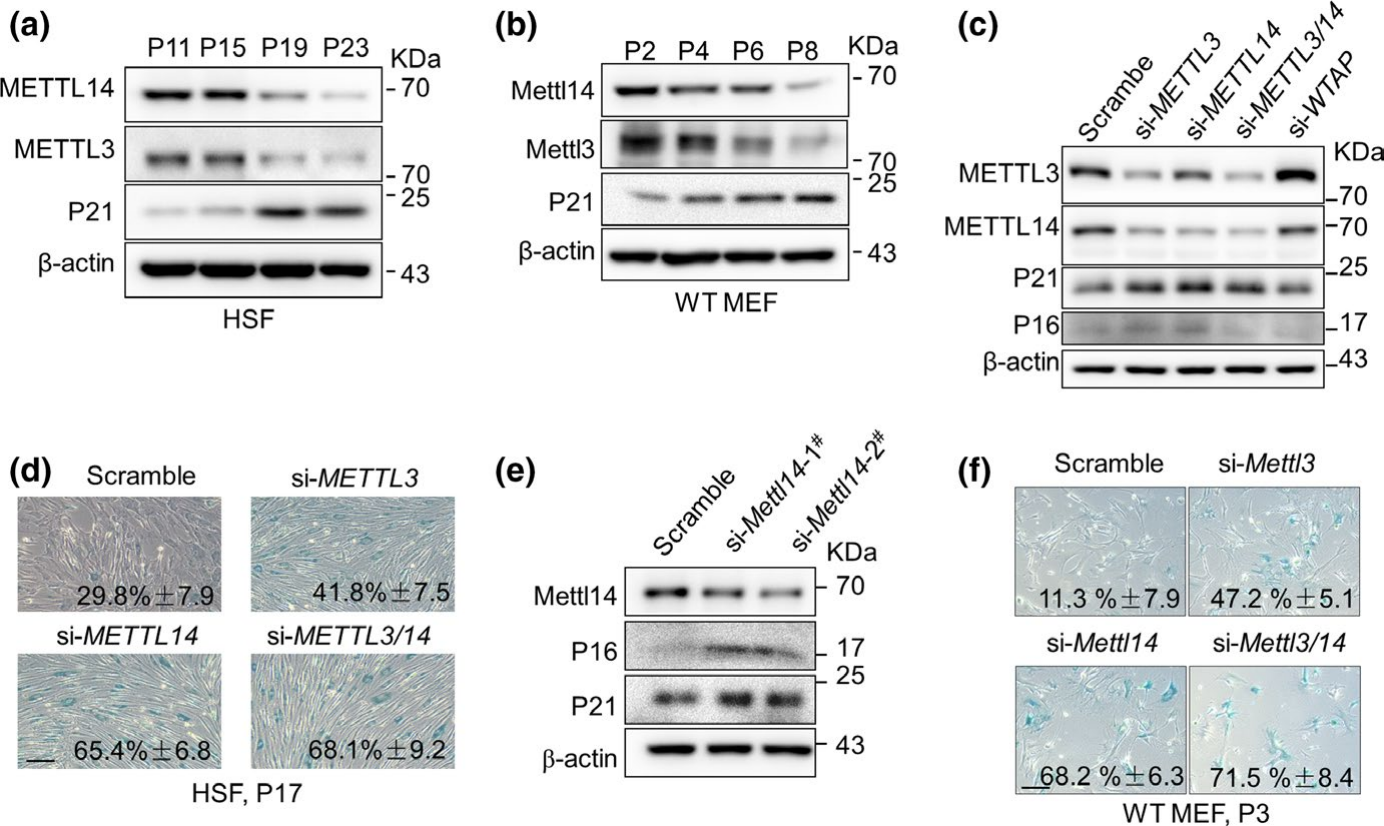
METTL3/14 decline accelerates senescence. (a, b) Western blot analysis of the METTL3/14 and p21^WIF1^ levels with subsequent passaging of human skin fibroblasts (HSFs) (a) and wild‐type (WT) mouse embryonic fibroblasts (MEFs) (b). (c) Western blot analysis of METTL3/14, p21, and p16 protein levels in HSFs (P17) treated with the indicated siRNAs. (d) Representative images of SA‐β‐Gal staining in HSFs (P17) treated with the indicated siRNAs. The percentage of SA‐β‐Gal‐positive cells is shown. The data represent the means ± *SEM*. Scale bar, 100 µm. (e) Western blot analysis of METTL14, p21, and p16 levels in MEFs (P3) treated with the indicated siRNAs. (f) Representative images of SA‐β‐Gal staining in WT MEFs treated with the indicated siRNAs. The percentage of SA‐β‐Gal‐positive cells is shown. The data represent the means ± *SEM*. Scale bar, 100 µm

During our analyses, we noticed that the protein levels of METTL3/14 declined with increasing passage of HGPS and healthy control cells (Figure 3d,e, P18 vs P15). As Progerin expression increases with passaging in human cells (Scaffidi & Misteli, 2006), we therefore asked whether METTL3/14 decline is a general feature of cellular senescence. Indeed, METTL3/14 expression levels were progressively reduced with passaging in both HSFs and MEFs (Figure 4a,b).

To examine whether METTL3/14 decline is a trigger or a consequence of senescence, we knocked down *METTL3*/*14* and examined the senescence status of both HSFs and MEFs. siRNA‐mediated *METTL3*/*14* knockdown (KD) led to a significant upregulation of p21^WIF1^ and p16^INK4A^ expression in HSFs (Figure 4c and Figure S5) and enhanced senescence‐associated β‐galactosidase activity (SA‐β‐gal) (Figure 4d). Similar results were obtained in MEFs. We thus conclude that METTL3/14 decline accelerates cell senescence.

### 2.5 METTL14 overexpression ameliorates cellular senescence

**FIGURE 5:**
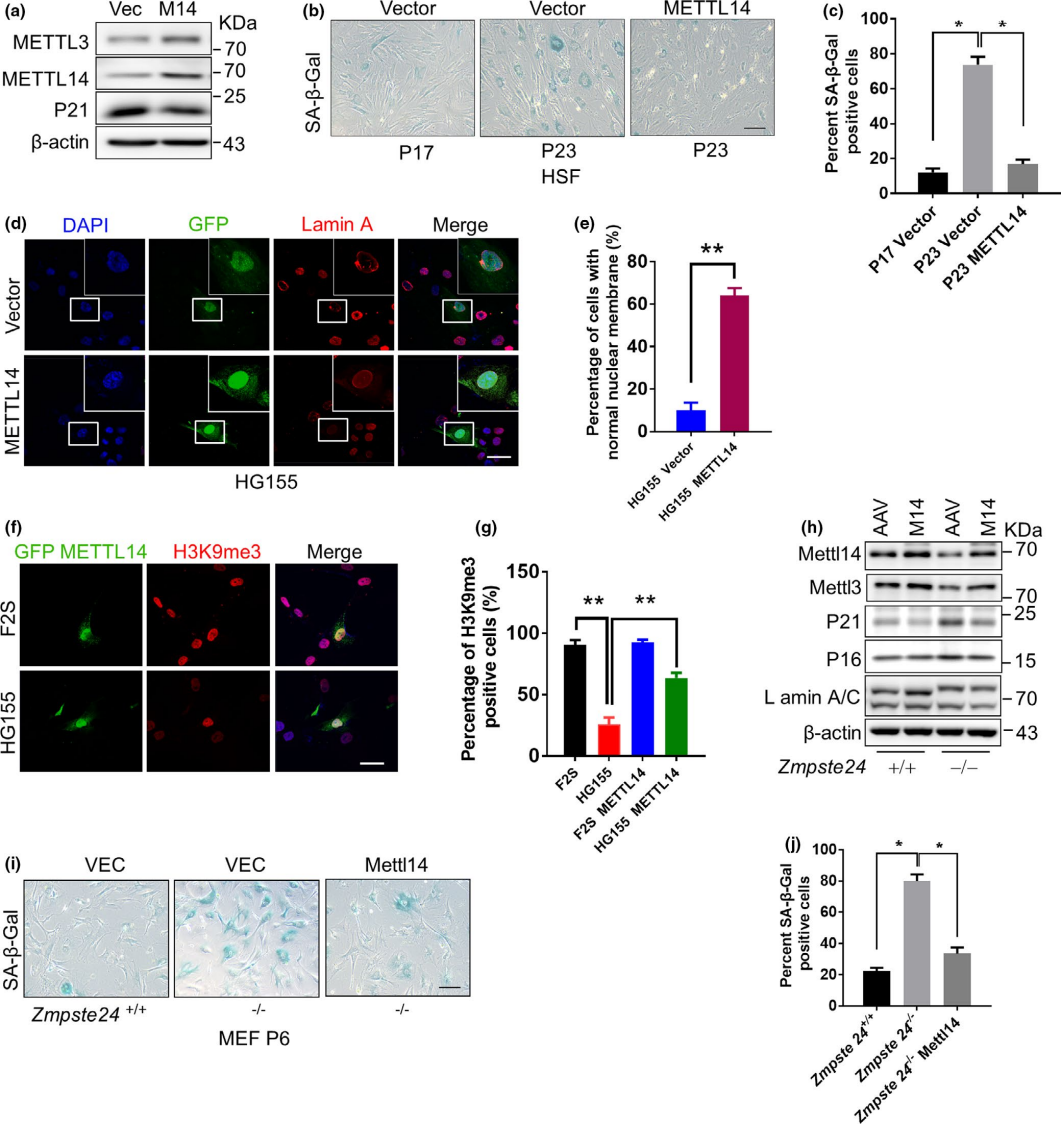
METTL14 overexpression ameliorates senescence. (a) Western blot analysis of METTL3/14 and p21 levels in lenti‐METTL14 (lenti‐M14)‐infected HSFs at P23. (b) Representative images of SA‐β‐Gal staining of lenti‐M14‐infected HSFs at the indicated passages. Scale bar, 100 µm. (c) Quantification of the β‐Gal‐positive cells in (b) based on 10 randomly chosen views for each group. The data represent the means ± *SEM*. *p < 0.05. (d) Representative immunofluorescence images of METTL14 and Lamin A expression in lenti‐METTL14‐infected HG155 at P23. Scale bar, 100 µm. (e) The percentage of cells with a normal nuclear shape (%) based on staining from 10 randomly chosen views for each group in (d). The data represent the means ± *SEM*. **p < 0.01. (f) Representative immunofluorescence images of METTL14 and H3K9me3 staining in lenti‐METTL14‐infected HG155 at P23. Scale bar, 100 µm. (g) Quantification of (f). The percentage of H3K9me3‐positive cells (%) based on 10 randomly chosen views for each group. The data represent the means ± *SEM*. **p < 0.01. (h) Western blot analysis of METTL3/14, p21, and p16 levels in adeno‐associated virus (AAV)‐METTL14‐infected *Zmpste24* ^−/−^ and *Zmpste24* ^+/+^ MEFs at P6. (i) SA‐β‐Gal staining of AAV‐METTL14‐infected *Zmpste24* ^−/−^ MEFs at P6. Scale bar, 100 µm. (j) Quantification of the SA‐β‐Gal‐positive cells in (i) from 10 randomly chosen views for each group. The data represent the means ± *SEM*. *p < 0.05. The data represent three independent experiments

In our final analyses, we asked whether *METTL14* overexpression could ameliorate senescence. To this end, we generated lentivirus particles overexpressing *METTL14* (lenti‐M14) and used them to infect HSFs at P23, when cell senescence phenotypes were obvious. METTL14 overexpression considerably reduced the level of p21 expression in the infected cells (Figure 5a). The percentage of SA‐β‐gal‐positive cells also dropped from an average of 72.13% in control cells to <20% in lenti‐M14‐infected HSFs (Figure 5b,c). Moreover, *METTL14* overexpression significantly alleviated nuclear membrane abnormalities (Figure 5d,e) and restored H3K9me3 levels (Figure 5f,g). We then tested the effect of AAV2/9‐mediated *METTL14* overexpression in *Zmpste24* ^−/−^ MEFs at P6. Here, we found that upon *METTL14* infection, p21 and p16 levels decreased (Figure 5h) and the SA‐β‐Gal level reduced (Figure 4i,j). Of note, the overexpression of *METTL14* merely affected the apoptotic level of HGPS cells compared with the empty vector control (Figure S6). Thus, *METTL14* overexpression seems to delay replicative senescence in normal fibroblasts and can rescue premature senescence in progeria.

## 3 DISCUSSION

Lamin A is extensively investigated as a component of nuclear lamina, and mounting evidences support its essential roles in regulating various nuclear activities such as nuclear shape, cargo transportation, chromatin structure, and gene expression (Prokocimer et al., 2009). Lamins are also constantly identified in multiple subnuclear domains; however, the related functions are rather overlooked. We found that Lamin A interacts with the m^6^A methyltransferase METTL3/14 in nuclear speckles. Lamin A deficiency compromises the nuclear speckle METTL3/14 reservoir and renders them susceptible to proteasome‐mediated degradation. Thus, Lamin A dictates a novel role of nuclear speckles and we propose a schematic model: Lamin A anchors METTL14 to the nuclear speckles via direct interaction, thus to safeguard METTL14 protein stability; without Lamin A, METTL14 loses its anchorage to the nuclear speckles and undergoes accelerated degradation (Figure S7). Future work remains to investigate how the Lamin A‐containing nuclear speckles protect METTL3/14 from proteasomal degradation. One possibility is that the compromised interaction between METTL3/14 and Lamins likely leaves it vulnerable by exposing ubiquitination sites.

The accumulation of abnormal Lamin A−prelamin A/Progerin disrupts nuclear lamina integrity, which is thought to be the cause of HGPS. Based on this rationale, treatment with farnesyltransferase inhibitors (FTIs) restore the nuclear structure abnormalities and alleviate premature aging in progeria murine models (Fong et al., 2006). The first clinical trial of FTI‒lonafarnib for HGPS treatment is now ongoing. However, only a subtle improvement of health status, reduction in mortality rate, and extension of life span (about 1‒2 years) were expected (Gordon et al., 2018). Thus, parallel mechanisms and additional treatment strategies are needed. Here, we for the first time demonstrate that abnormal Lamin A not only jeopardizes the nuclear shape, but also compromises subnuclear domains exemplified by the nuclear speckles. Consequently, METTL14 is significantly downregulated in HGPS cells and the late passage of normal cells. Most importantly, *METTL14* overexpression attenuates senescence in both normal cells and HGPS cells. Indeed, it has been shown that the alternative splicing of *LMNA* exon 11 may also occur in normally senescent cells, which causes the accumulation of Progerin (Scaffidi & Misteli, 2006). It is thus speculated that the accumulation of Progerin might render METTL14 susceptible to proteasomal degradation during replicative senescence.

In summary, Lamin A safeguards the proper localization of METTL3/14 in nuclear speckles. Abnormal Lamin A mislocalizes METTL3/14 from the nuclear speckles and induces proteasome‐mediated degradation. The data reveal a novel mechanism by which Lamin A maintains the METTL3/14 reservoir and highlights the importance of m^6^A RNA methylation in senescence.

## 4 MATERIALS AND METHODS

### 4.1 Cell lines and cell culture

Human primary dermal fibroblast cells HGADFN122 and HGADFN155 were obtained from the Progeria Research Foundation (PRF). Primary mouse embryonic fibroblasts (MEFs) and human skin fibroblasts HSFs (F2‐S) were prepared as previously described (Liu et al., 2005). *Zmpste24* ^−/−^ MEFs were isolated from 13.5‐day‐old embryos of *Zmpste24* ^+/−^ intercrossed mice (C57BL6). Littermate‐matched *Zmpste24* ^+/+^ and *Zmpste24* ^−/−^ MEFs were cultured in Dulbecco's modified Eagle medium (DMEM) containing 10% fetal bovine serum (FBS). *Lmna* ^−/−^ MEFs were obtained from *Lmna* ^−/−^ mice. Primary MEFs and human F2S fibroblasts were maintained in DMEM (Gibco) supplemented with 4.5 g/L glucose, 1% Pen Strep, and 15% FBS. HEK293 (CRL‐1573) cells were purchased from ATCC.

### 4.2 Cell transfection and RNA interference

Plasmid transfections were performed using Lipofectamine^®^ 3000 (Invitrogen) and siRNA transfections were performed using Lipofectamine^®^ RNAiMAX (Invitrogen), following the manufacturer's instructions. Specific custom siRNAs were synthesized by GenePharma. The siRNA sequences are listed in Table S1.

### 4.3 Plasmids construction

The human METTL3/14 gene was cloned into a pcDNA3‐FLAG vector (Addgene) and then subcloned into pKH3‐HA, pEGFP‐C1, DsRedC1, and pET32a vectors (Addgene). The primer sequences used for cloning are listed in Table S1.

### 4.4 Antibodies

The following antibodies were used in this study: anti‐HA (ab9110), anti‐FLAG (ab1162), anti‐SC‐35 (ab11826), anti‐H3K9me3 (ab8898), anti‐METTL3 (ab66660), and anti‐p16 (ab54210) were purchased from Abcam; anti‐METTL14 (PA5‐58204) was from Thermo Fisher Scientific; anti‐mouse IgG‐Cy3, anti‐rabbit IgG‐Cy3, anti‐mouse IgG‐FITC, anti‐rabbit IgG‐FITC, anti‐FLAG M2 affinity gel, and monoclonal anti‐HA agarose were from Sigma‐Aldrich; anti‐Lamin A/C (sc‐20681), anti‐Lamin A (sc‐518013), anti‐Lamin A (sc‐71481), anti‐p21 (sc‐6246), and anti‐p16 (ab54210) were from Santa Cruz Biotechnology; anti‐m^6^A (No. 202003) was from Synaptic Systems; rabbit anti‐γH2AX (05‐636) was from EMD Millipore; rabbit anti‐HMGB1 (#3935), anti‐Ubiquitin (#3936), and GAPDH (14C10) mAb (#2118) were from Cell Signaling Technology; and mouse/rabbit Alexa Fluor 488/Alexa Fluor 594 was from Thermo Fisher Scientific.

### 4.5 Western blotting and co‐immunoprecipitation

Cell extracts for Western blotting were lysed in buffer (20 mM Tris‐HCl [pH 7.5], 0.1 M NaCl, 0.5% NP‐40, 1 mM EDTA, 1 mM DTT, and a cOmplete protease inhibitor cocktail [Roche]), boiled in sodium dodecyl sulfate (SDS) sample loading buffer, resolved by SDS‐PAGE, and transferred to PVDF membranes (Millipore). The membranes were blocked in 5% milk in Tris‐buffered saline and Tween 20 (TBST: 150 mM NaCl, 20 mM Tris‐HCl [pH 7.6], 0.05% Tween 20) for 1 h at room temperature and subjected to immunoblotting with the indicated antibodies overnight at 4°C. The membranes were then probed with the respective secondary antibodies linked to HRP for 1 h at room temperature. Immunoreactive products were visualized using an Enhanced Chemiluminescence Kit (Pierce) and a Bio‐Rad imaging system.

For immunoprecipitation, the cells were exposed to the indicated treatments and then harvested in lysis buffer (20 mM Tris‐HCl [pH 7.5], 300 mM NaCl, 10% glycerol, 0.1 mM EDTA, 0.1% NP‐40, and a cOmplete protease inhibitor cocktail). The cell lysates were incubated with 1 µg of the respective antibodies or control IgGs, and then were cross‐linked to protein A/G agarose beads at 4°C overnight with rotation. The bead‐bound immunoprecipitates were washed with lysis buffer, and the beads were boiled in SDS sample loading buffer. The inputs and immunoprecipitated products were analyzed by Western blotting.

### 4.6 Immunofluorescence staining

Cells were fixed with 4% paraformaldehyde on ice and permeabilized with PBS containing 0.1% Triton X‐100 for 15 min. Then, the cells were blocked with 1% bovine serum in PBS for 30 min at room temperature. The coverslips were first incubated with primary antibody overnight at 4°C and then detected by Alexa Fluor‐conjugated secondary antibodies (Alexa 488, Alexa 594; 1:500, Life Technologies) for 1 h at room temperature in the dark and mounted with DAPI‐containing mounting medium. Images were captured under an immunofluorescence confocal microscope (Zeiss). A representative image for each condition is shown.

### 4.7 RNA isolation and quantitative RT‐PCR

Total RNA was isolated with TRIzol^®^ reagent (Invitrogen) from WT or transiently transfected cells. DNase I‐treated total RNA was used to synthesize cDNA using an iScript cDNA Synthesis Kit (Bio‐Rad) according to the manufacturer's protocols. Real‐time quantitative polymerase chain reaction (RT‐qPCR) was performed using 2 × SYBR Green Mix (Takara) in a Bio‐Rad detection system. Each sample was run in triplicate, and the gene expression levels were normalized to β‐actin. The primer sequences are listed in Table S1.

### 4.8 Senescence‐associated β‐gal assay

Senescence‐associated β‐galactosidase (SA‐β‐gal) activity was determined using a Cellular Senescence Assay Kit (Cell signaling). Briefly, cells were seeded in 6‐well plates, fixed with 4% paraformaldehyde at room temperature for 15 min, and then washed with 1× PBS, stained with 2 ml freshly prepared 1× β‐gal detection solution at 37°C overnight in the dark. The cells were washed twice with 1× PBS, overlaid with 70% glycerol/PBS, and images were captured under a microscope. The number of blue‐stained cells was counted from >250 randomly chosen cells. The data were analyzed by two‐tailed Student's *t* test.

### 4.9 m^6^A dot blotting

mRNA was denatured at 75°C for 5 min, spotted, and cross‐linked to a positively charged nylon membrane 2× in UV Stratalinker with 1800 μJ/cm^2^ at 254 nm. The membrane was probed with m^6^A antibody (No. 202003, 1:1000; Synaptic Systems) overnight at 4°C and then incubated with goat anti‐rabbit IgG‐HRP (1:10,000 dilution) in 10 ml dilution buffer for 1 h at room temperature with gentle shaking. After washing four times, immunoreactive products were visualized using an Enhanced Chemiluminescence Kit (Pierce) and a Bio‐Rad imaging system.

### 4.10 Statistical analyses

Statistical analyses were performed in GraphPad Prism 7 (GraphPad Software Inc., USA) using a two‐tailed *t* test. Statistical significance was considered as *p < 0.05, **p < 0.01, and ***p < 0.001. The data represent the means ± *SEM* of three independent experiments.

## CONFLICT OF INTEREST

The authors declare no conflict of interest.

## AUTHOR CONTRIBUTIONS

J.Z. and Y.A. conducted the experiments; Z.Z., Y.M., L.P., and Y.J. provided technical support and analyzed data; and J.Z., Z.W., and B.L. designed the study and wrote the manuscript.

## Supporting information

Supplementary Material

Table S1

## Data Availability

The data that support the findings of this study are available from the corresponding author upon reasonable request.
