# Supplementary material for: Lamin A safeguards the m6A methylase METTL14 nuclear speckle reservoir to prevent cellular senescence
Source: Aging Cell. 2020 Aug 19;19(10):e13215. doi: 10.1111/acel.13215 (PMC7576246; doi:10.1111/acel.13215)
Supplement: Supplementary file 1 — Supplementary Material [file ACEL-19-e13215-s001.pdf]

Supplementary Figures

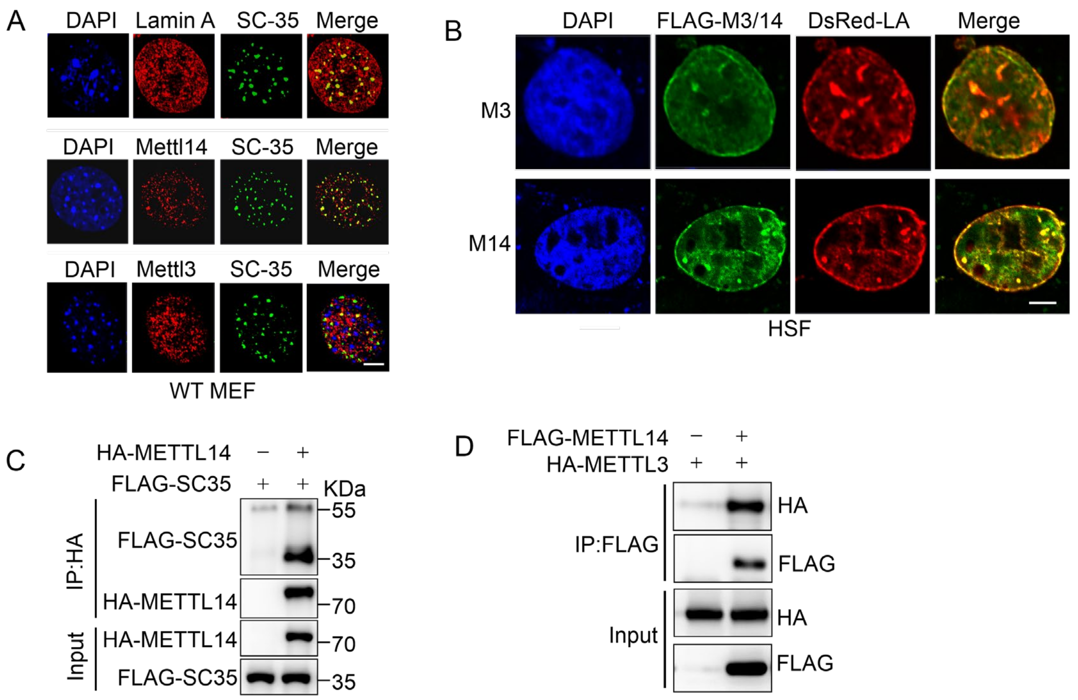

Figure S1. Co-localization of Lamin A and METTL3/14 in the nuclear speckles

(**A**) Representative images of immunofluorescence staining of SC-35 (red), Lamin A (green) and METTL3/14 (red) in wild-type (WT) mouse embryonic fibroblasts (MEFs). Scale bar, 5 µm. (**B**) Representative images of the confocal microscopy analysis of DsRed-Lamin A (DsRed-LA) and FLAG-METTL3/14 (FLAG-M3/14) expression in human skin fibroblasts (HSFs). Scale bar, 5 µm. (**C-D**) Co-immunoprecipitation (Co-IP) and western blot analysis of the interactions between HA-METTL4 and FLAG-SC35 (**C**); FLAG-METTL14 and HA-METTL3 (**D**) in HEK293 cells.

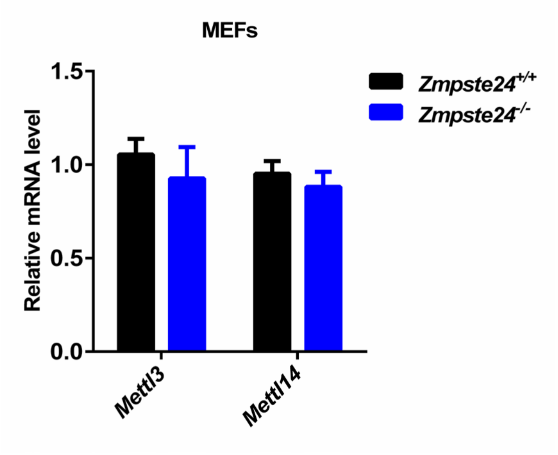

Figure S2. ***Mettl3/14*** **RNA levels in** ***Zmpste24*** $^{-/-}$ **MEFs**

Quantitative RT-PCR analysis of *Mettl3/14* mRNA levels in *Zmpste24* $^{-/-}$ MEFs and wild-type littermate control (*Zmpste24* $^{+/+}$*)* MEFs at passage 4. The data represent the means ± s.e.m.

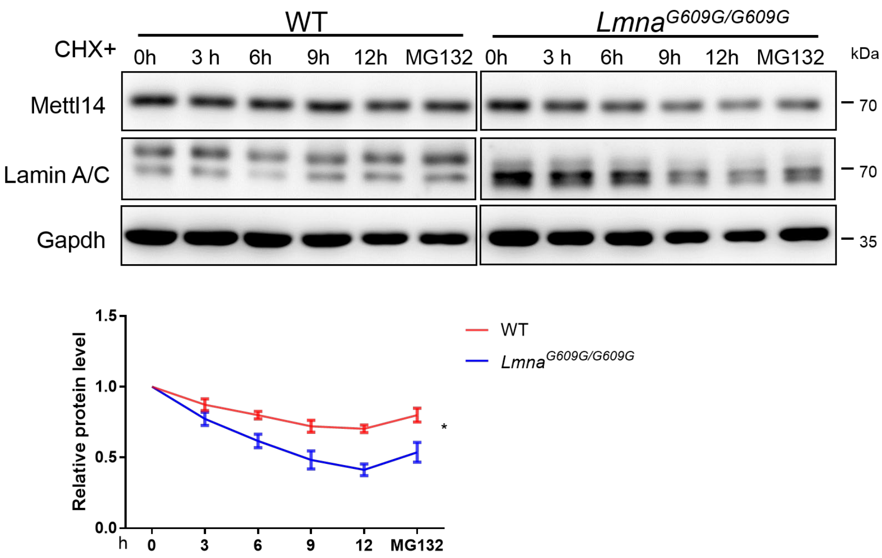

Figure S3. The degradation of METTL14 in ***Lmna*** $^{G609G/G609G}$ **MEFs**

Upper, representative immunoblots showing Mettl14 protein levels in *Lmna* $^{G609G/G609G}$ MEFs and wild-type (WT) controls in the presence of CHX (50 μg/ml) or MG132. Lower, quantification of Mettl14 levels was performed by Image J®. The data represent the means ± s.e.m. *$p < 0.05$.

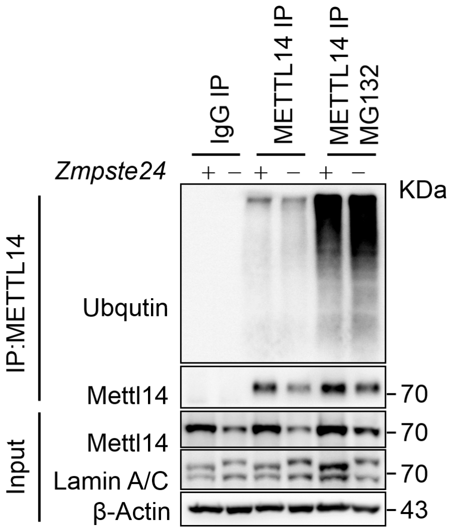

Figure S4. Ubiquitination level of METTL14

Western blot analysis of METTL14 ubiquitination in *Zmpste24*$^{-/-}$ MEFs and wild-type littermate control (*Zmpste24*$^{+/+}$) MEFs. The cells were treated with MG132 (MG) for 6 h and METTL14 was pulled down in the anti-METTL14 immunoprecipitates.

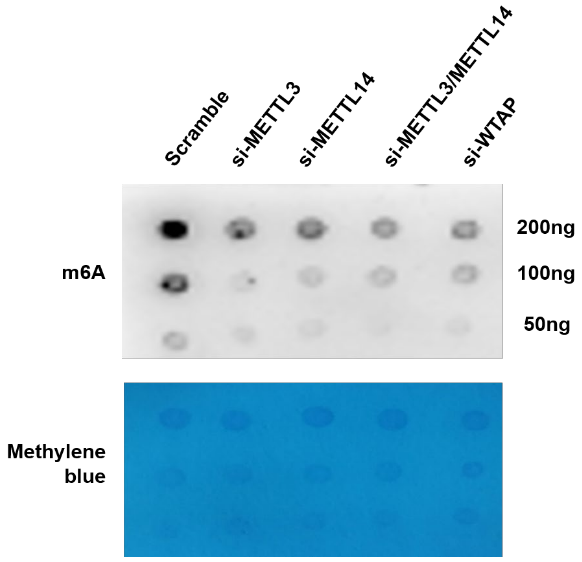

Figure S5. METTL3/14 knockdown downregulates m^{6}A level.

Dot blot showing m^{6}A levels in cells treated with Scramble or si-METTL3, si-METTL14, siMETTL3/14 and si-WTAP siRNAs in human skin fibroblasts (HSFs) at P17.

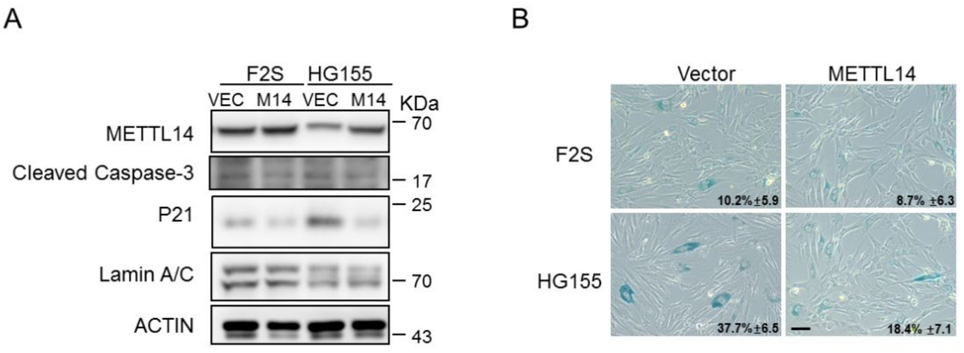

Figure S6. ***METTL14*** **overexpression merely changed the apoptotic level of HGPS cells**

(A) Representative immunoblots showing the levels of cleaved caspase-3 (cCasp-3) and P21 in Lenti-M14-infected HGPS 155 or F2S controls at the passages 18. (B) Senescence-associated β-galactosidase staining in Lenti-M14-infected HGPS 155 or F2S controls at the passages 18 (scale bar, 200 μm).

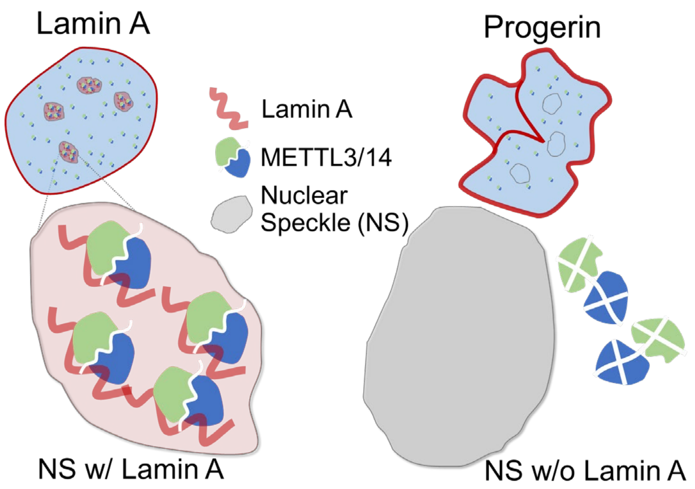

Figure S7. A schematic model

Lamin A safeguards METTL14 reservoir in the nuclear speckles; abnormal Lamins mis-localize METTL14 from the nuclear speckles leading to protein degradation.
